# First record of *Phanuropsis laniger* Johnson in Brazil and first record of *Phanuropsis semiflaviventris* Girault in Amazonas (Hymenoptera: Platygastridae) with notes of their hosts, stink-bugs of cupuaçu

**DOI:** 10.3897/BDJ.4.e8142

**Published:** 2016-04-14

**Authors:** Thiago Mahlmann, Bruno G Oliveira

**Affiliations:** ‡ Instituto Nacional de Pesquisas da Amazônia, Manaus, Brazil

**Keywords:** *
Antiteuchus
*, Parasitoid, Telenominae, *Theobroma grandiflorum*

## Abstract

**Background:**

We report the first record and collection of *Phanuropsis laniger* Johnson, 1987, for Brazil since its original description. In addition we expand the distribution of *Phanuropsis semiflaviventris* Girault, 1916, for Amazonas, Brazil and present an updated list of records for both species with the occurrence of *P. semiflaviventris* in egg of stink-bugs on cupuaçu (*Theobroma grandiflorum*).

**New information:**

First record of *Phanuropsis laniger* Johnson, 1987, for Brazil. We also expanded the distribution of *Phanuropsis semiflaviventris* Girault, 1916, for the state of Amazonas, Brazil

## Introduction

The Neotropical genus *Phanuropsis* Girault (Hymenoptera: Platygastridae) is represented by two species: *Phanuropsis semiflaviventris* Girault, 1916, and *Phanuropsis laniger* Johnson, 1987 (Hymenoptera Online, various contributors 2016). The most common species is *P. semiflaviventris* which can be found from Belize to southern Brazil (Oliveira 2016). *Phanuropsis laniger* is rarely collected, until now known only from two localities in Colombia and Peru.

Host associations are known for both species (Hymenoptera Online, various contributors 2016) in which *P. semiflaviventris* was found in association mainly with *Antiteuchus* Dallas (Hemiptera: Pentatomidae) and *P. laniger* was associated only with *Edessa* Fabricius (Hemiptera: Pentatomidae). Relationships with species and their hosts can support additional information on behavior from species also facilitating future collections.

The objective of this work was to report the first occurrence of *P. laniger* in Brazil with the first collection after its description. In addition we report the first record of *P. semiflaviventris* for Amazonas, Brazil updating the occurrence with some notes on the parasitism in eggs of stink-bugs on “cupuaçu” (*Theobroma grandiflorum*)

## Materials and methods

The collections were taken at different times in 2014-2015 at natural vegetation areas containing "cupuaçu trees" (*Theobroma grandiflorum*) of the Instituto Nacional de Pesquisas da Amazônia (INPA), located in the city of Manaus, Amazonas, Brazil (3°5'45.80"S; 59°59'21.86"W). Cupuaçu trees canopies were sampled randomly seeking for ovipositions of Hemiptera. Egg masses with signs of parasitism were collected and held in an incubator with temperature of 25ºC ±1, humidity of 70% ±5 and photo period of 12 hours of light and 12 hours of dark until the emergence of adults. After the emergency the insects were killed and mounted for identification. The specimens of *Phanuropsis* were identified based on the diagnosis of the genera and by the discussion presented by Johnson 1987). Photos were sent to Lubomir Masner to confirm our identification.

Photomicrographs were prepared using a LeicaM205C stereomicroscope coupled with a Leica DFC295 and a Leica Application Suite V4.1 Interactive Measurements, Montage. All the material is deposited at Invertebrates Collection of INPA.

## Taxon treatments

### Phanuropsis laniger

Johnson, 1987

http://hol.osu.edu/index.html?id=2577

#### Distribution

This species is so far known from Colombia and Peru (Johnson 1987) and is a new record for Brazil (Table 1).

### Phanuropsis semiflaviventris

Girault, 1916

http://hol.osu.edu/index.html?id=2578

#### Distribution

This species is so far known from Central America (Honduras, Costa Rica and Panama) and South AmericaI (Colombia, Venezuela, Trinidad and Tobago, Suriname and Brazil). In Brazil is a new record for Amazonas (Table 1​).

## Discussion

A single female specimen of *P. laniger* was collected during the period of observation (Fig. 1). The insect was at the bottom of a cupuaçu leaf near an egg mass of *Antiteuchus* sp. We did not observe however the contact of *P. laniger* with those eggs even though they were collected at the exact time of observation. This is the first record of *P. laniger* in Brazil (Table 1) with the first record of collection since its original description in 1987.

Were collected seven Hemiptera egg postures in the cupuaçu trees all belonging to genus *Antiteuchus*. The species of parasitoids emerged were *Gryon* sp., *P. semiflaviventris* (Fig. 2) and *Trissolcus urichi* Crawford. This is the first record of *P. semiflaviventris* in the Amazonas, Brazil. Thus, there are currently records for the North, Northeast and Southeast of Brazil.

## Supplementary Material

Supplementary material 1 Phanuropsis laniger_DarwinCoreFile Data type: occurences
File: oo_82421.xlsx Mahlmann, Olivera & Elijah

Supplementary material 2 Phanuropsis semiflaviventris_DarwinCoreFile Data type: occurences
File: oo_82422.xlsx Mahlmann, Oliveira & Elijah

XML Treatment for Phanuropsis laniger

XML Treatment for Phanuropsis semiflaviventris

## Figures and Tables

**Figure 1a.**
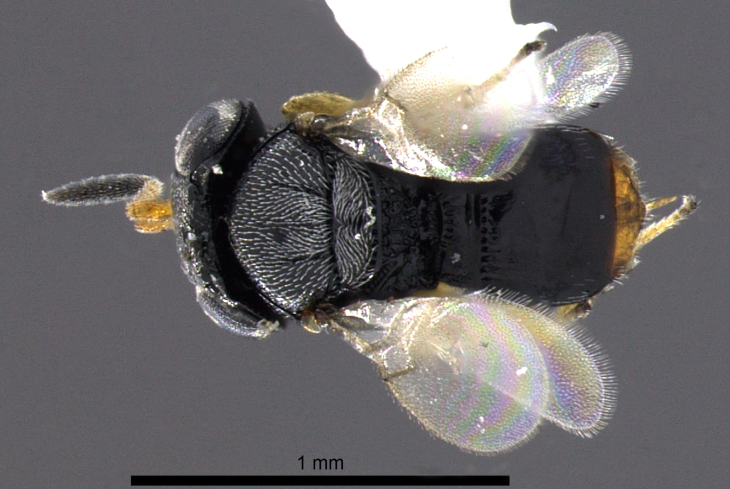
Dorsal view

**Figure 1b.**
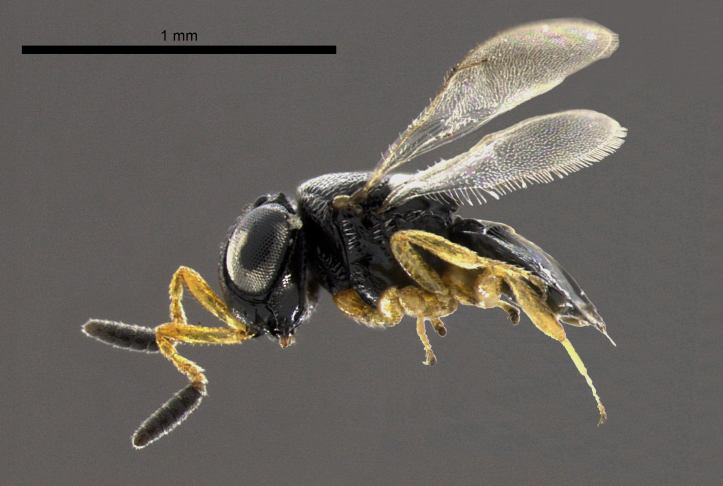
Lateral view

**Figure 2a.**
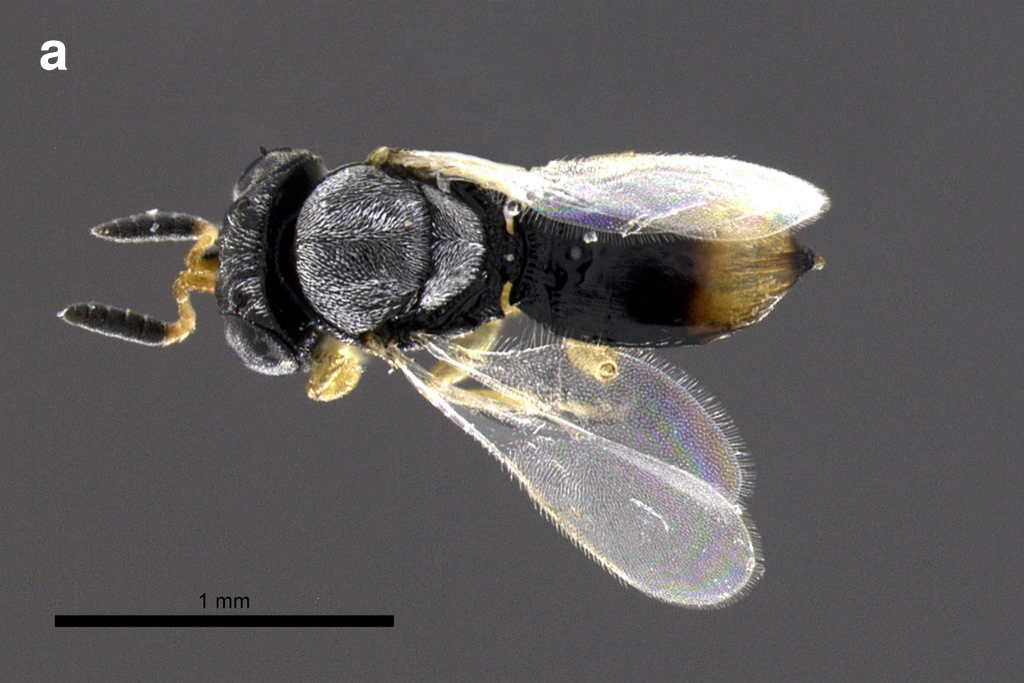
Dorsal view

**Figure 2b.**
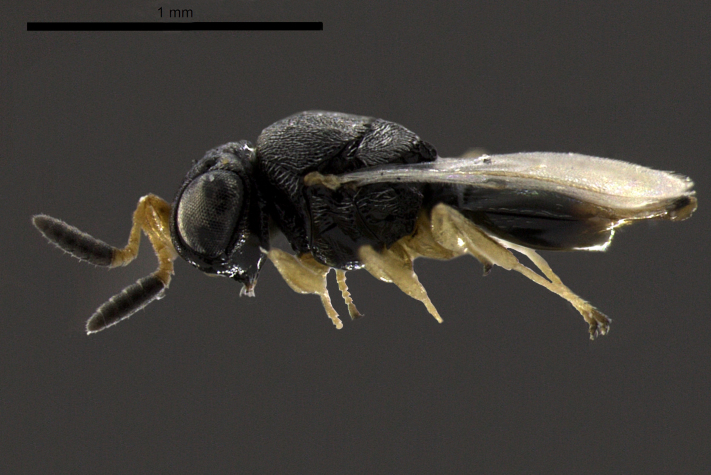
Lateral view

**Table 1.** List of species of *Phanuropsis* Girault occurring in Brazil, including regions and the states where they were collected.

**Species**	**Region**	**Locality**	**Reference**
*P. semiflaviventris*	North	Amazonas*	Mahlmann & Oliveira (present study)*
		Pará	Hymenoptera Online, various contributors (2016)
	Northeast	Pernambuco	Loiácono and Margaría (2002)
		Piauí	Paz-Neto et al. (2015)
	Southeast	Espírito Santo	Azevedo et al. 2015
		Rio de Janeiro	Santos and Albuquerque (2001)
		São Paulo	Johnson 1987
*P. laniger*	North	Amazonas*	Mahlmann & Oliveira (present study)*
* new record			
